# Hyaluronan Esters Drive Smad Gene Expression and Signaling Enhancing Cardiogenesis in Mouse Embryonic and Human Mesenchymal Stem Cells

**DOI:** 10.1371/journal.pone.0015151

**Published:** 2010-11-30

**Authors:** Margherita Maioli, Sara Santaniello, Andrea Montella, Pasquale Bandiera, Silvia Cantoni, Claudia Cavallini, Francesca Bianchi, Vincenzo Lionetti, Flavio Rizzolio, Irene Marchesi, Luigi Bagella, Carlo Ventura

**Affiliations:** 1 Department of Biomedical Sciences, University of Sassari, Sassari, Italy; 2 National Institute of Biostructures and Biosystems, University of Bologna, Bologna, Italy; 3 Laboratory of Molecular Biology and Stem Cell Engineering, Cardiovascular Department, National Institute of Biostructures and Biosystems, S. Orsola - Malpighi Hospital, University of Bologna, Bologna, Italy; 4 Sector of Medicine, Scuola Superiore Sant 'Anna, Pisa, Italy; 5 Sbarro Institute for Cancer Research and Molecular Medicine, Center for Biotechnology, Temple University, Philadelphia, Pennsylvania, United States of America; 6 Bioscience Institute, Falciano, Republic of San Marino; Seattle Children's Research Institute, United States of America

## Abstract

**Background:**

Development of molecules chemically modifying the expression of crucial orchestrator(s) of stem cell commitment may have significant biomedical impact. We have recently developed hyaluronan mixed esters of butyric and retinoic acids (HBR), turning cardiovascular stem cell fate into a high-yield process. The HBR mechanism(s) remain still largely undefined.

**Methodology/Principal Findings:**

We show that in both mouse embryonic stem (ES) cells and human mesenchymal stem cells from fetal membranes of term placenta (FMhMSCs), HBR differentially affected the patterning of Smad proteins, one of the major conductors of stem cell cardiogenesis. Real-time RT-PCR and Western blot analyses revealed that in both cell types HBR enhanced gene and protein expression of Smad1,3, and 4, while down-regulating Smad7. HBR acted at the transcriptional level, as shown by nuclear run-off experiments in isolated nuclei. Immunofluorescence analysis indicated that HBR increased the fluorescent staining for Smad1,3, and 4, confirming that the transcriptional action of HBR encompassed the upregulation of the encoded Smad proteins. Chromatin immune precipitation and transcriptional analyses showed that HBR increased the transcription of the cardiogenic gene Nkx-2.5 through Smad4 binding to its own consensus Smad site. Treatment of mouse ES cells and FMhMSCs with HBR led to the concomitant overexpression of both Smad4 and α-sarcomeric actinin. Smad4 silencing by the aid of lentiviral-mediated Smad4 shRNA confirmed a dominant role of Smad4 in HBR-induced cardiogenesis.

**Conclusions/Significance:**

The use of HBR may pave the way to novel combinatorial strategies of molecular and stem cell therapy based on fine tuning of targeted Smad transciption and signaling leading to a high-throughput of cardiogenesis without the needs of gene transfer technologies.

## Introduction

Embryonic stem (ES) cells differentiate *in vitro* into various cell types including cardiac myocytes [1], [2], by tracing or mimicking developmental processes occurring *in vivo*. Moreover, ES cells besides being a tool to investigate the molecular mechanisms underlying cardiogenesis, represent a source in regeneration therapies for damaged tissues [3]. Although ES cells have unlimited replication capacity, the cardiogenic process is inefficient, with only a limited number of cells becoming cardiac myocytes [4]. Consequently, developing new molecules that maximize the functional expression of crucial signaling orchestrator(s) of targeted commitments within a population of precursor cells may offer significant advantages in tissue repair and clinical therapies. These molecules may also provide new insights into the dynamic circuitry underlying developmental decisions.

We have developed a mixed ester of hyaluronan with butyric and retinoic acids (HBR), acting as a differentiating agent, remarkably increasing the yield of cardiomyocytes derived from mouse ES cells [4]. This compound was also effective as a tool implementing the cardiogenic process in human mesenchymal stem cells (hMSCs) isolated from the bone marrow and alternative sources, including the dental pulp and fetal membranes of term placenta (FMhMSCs) [5]. The cardiogenic program induced by HBR was due to the transcriptional induction of two key regulators of cardiogenic commitment, GATA-4 and Nkx-2.5, early expressed during heart formation [6], [7]. HBR also enhanced prodynorphin gene transcription and the expression of its biologically active peptide, the k opioid receptor agonist dynorphin B, both being directly implicated in cardiac lineage specification [8]–[11].

The Smad proteins are a family of signal transducers which can be divided into three distinct subfamilies: receptor-regulated Smads (R-Smads), common-partner Smads (Co-Smads) and inhibitory Smads (I-Smads) [12], [13]. Compelling evidence suggests that a Smad signaling pathway is important in stem cell cardiovascular differentiation. In vertebrates and *Drosophila*, heart formation is an early event occurring in the embryo, which requires the activation of a bone morphogenetic protein (BMP) signaling, representing a part of the transforming growth factor beta (TGF-β) superfamily of growth factors [14]–[16]. Activated BMP receptor kinases specifically and transiently interact with and phosphorylate particular R-Smads. Subsequently, the activated R-Smads recruit Co-Smads and heteromeric complexes accumulate in the nucleus [12], [13]. Nkx-2.5 is the earliest known marker of the cardiac lineage, and is expressed together with the cardiogenic commitment of cells from anterior-lateral mesoderm [17], [18]. Its expression is maintained in the heart throughout prenatal and postnatal life. In *Drosophila*, Smad4 and R-Smads (Smad1, Smad5, and Smad8), may regulate Nkx-2.5 gene expression via a Smad-binding domain located in its promoter region [19]. Moreover, mouse Smad4 mutant embryos [20] show significant reduction of Nkx-2.5 expression in prospective cardiac mesoderm, confirming previous findings, indicating a requirement for Smad1/4 in the activation of Nkx-2.5 gene transcription [21].

In the current study, the gene and protein expression profile of Smad1,3,4, and 7 were investigated for different periods of time in both mouse ES cells and FMhMSCs exposed to HBR. Our data provide evidence that the mixed ester is able to drive a complex circuitry of Smad transcription and signaling that is essential in stem cell cardiogenesis.

## Results

### HBR Modulates the Gene Expression of Smad1,3,4, and 7 in GTR1 ES Cells and FMhMSCs

GTR1 cells [9], [11], a derivative of R1 ES cells bearing the puromycin-resistance gene driven by the cardiomyocyte-specific α-myosin heavy chain promoter, were used as an *in vitro* model of cardiogenesis to elucidate whether a recruitment of Smad signaling may be a mechanism underlying the cardiogenic commitment induced by HBR.

When cultured in the absence of LIF and in suspension, these cells aggregate in EBs, evolving into spontaneously beating cardiomyocytes throughout 7–8 days of culturing. After puromycin selection, a virtually pure population of ES-derived cardiomyocytes can be obtained.

The gene expression of Smad1,3,4, and 7 was investigated by real-time RT-PCR at different times of GTR1 ES culture in the absence or presence of HBR. To further investigate whether HBR may have a role in Smad patterning, the hyaluronan mixed ester was also applied to FMhMSCs, a hMSC population that due to HBR treatment could be committed to the cardiovascular lineage *in vitro*, affording remarkable myocardial repair *in vivo* after transplantation in infarcted rat hearts [5]. In both cell types, HBR was used at a concentration of 1.5 mg/ml that was previously shown to afford a maximal cardiogenic response [4], [5].

**Figure 1:**
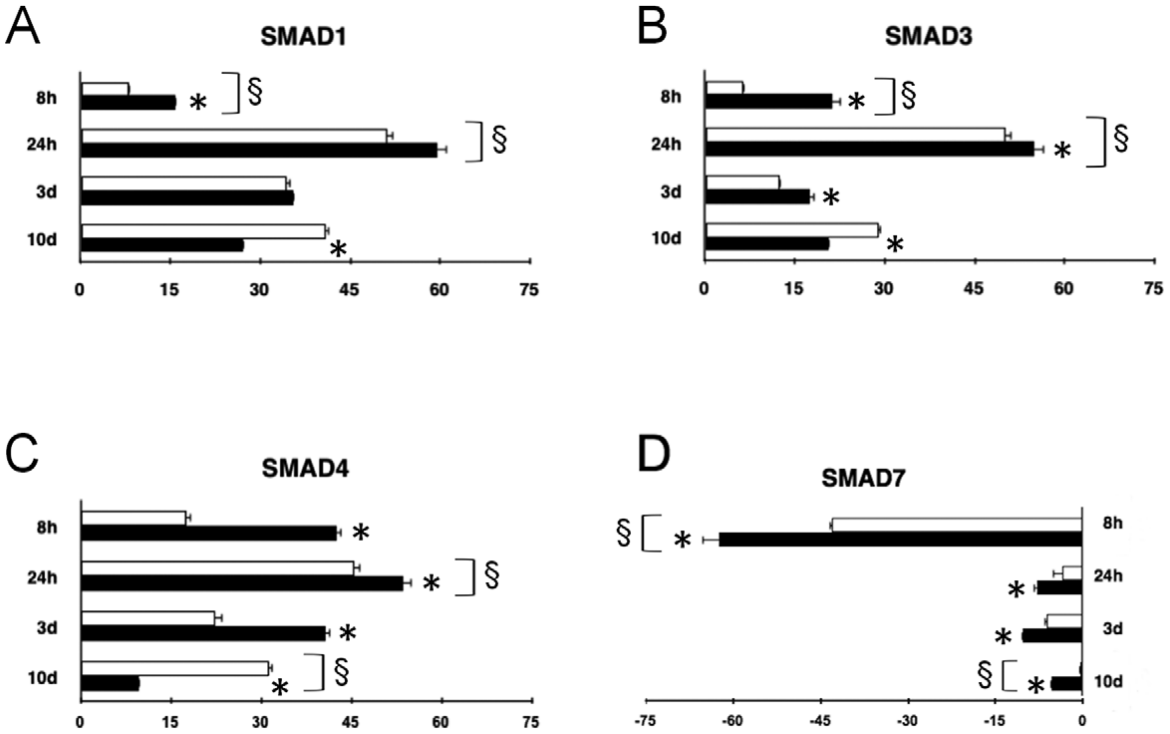
Time-course of Smad gene expression in GTR1-derived embryoid bodies and in puromycin-selected cardiomyocytes. Cells were cultured for 8, 24 hours, 3 days (EBs), or 10 days (puromycin-selected cardiomyocytes) in the absence or presence of HBR (1.5 mg/ml). The amount of each Smad, Smad1 (A), Smad3 (B), Smad4 (C) and Smad7 (D) mRNA from untreated (white bars) or HBR-treated (black bars) cells was normalized to GAPDH and was plotted as fold change relative to the mRNA expression at the time of LIF removal (time 0), defined as 1 (mean ± S.E.; n = 6). *, significantly different from untreated group;§, significantly different from the other groups (one-way analysis of variance, Tukey's test).

After 8 hours of culture, following LIF withdrawal (time zero), EBs consistently exhibited Smad1,3 and 4 gene expression (Figure 1A–1C). HBR-treated EBs revealed a remarkably higher gene expression of each isoform, as compared to untreated cells. At this time point, Smad7 mRNA declined compared to time zero, and was further significantly downregulated in the presence of HBR (Figure 1D).

After 24 hours, the gene expression of Smad1-4 increased compared to time zero, retaining higher levels in HBR-treated than untreated cells (Figure 1A–1C). The spontaneous gene expression of these Smads declined after 72 hours of culture, with Smad4, and to a lesser extent Smad3, still exhibiting higher mRNA levels in HBR-treated than in unexposed cells (Figure 1B, 1C). On the contrary, at this time point, Smad1 was similarly expressed in both groups of cells (Figure 1A).

Comparative analysis of Smad1,3, and 4 mRNA was also performed at 10 days in ES-derived cardiomyocytes recovered after exposure in the absence or presence of HBR from the time of LIF removal throughout 4 days of puromycin selection. Under these experimental conditions, HBR reversed its effect, downregulating the gene expression level, as compared to unexposed controls (Figure 1A–1C).

The gene expression of Smad7 progressively increased in EBs after 24 hours of culture, approaching the time zero level after 10 days in ES-derived cardiomyocytes, but remaining downregulated in HBR-exposed cells, as compared with the corresponding untreated time control (Figure 1D).

**Figure 2:**
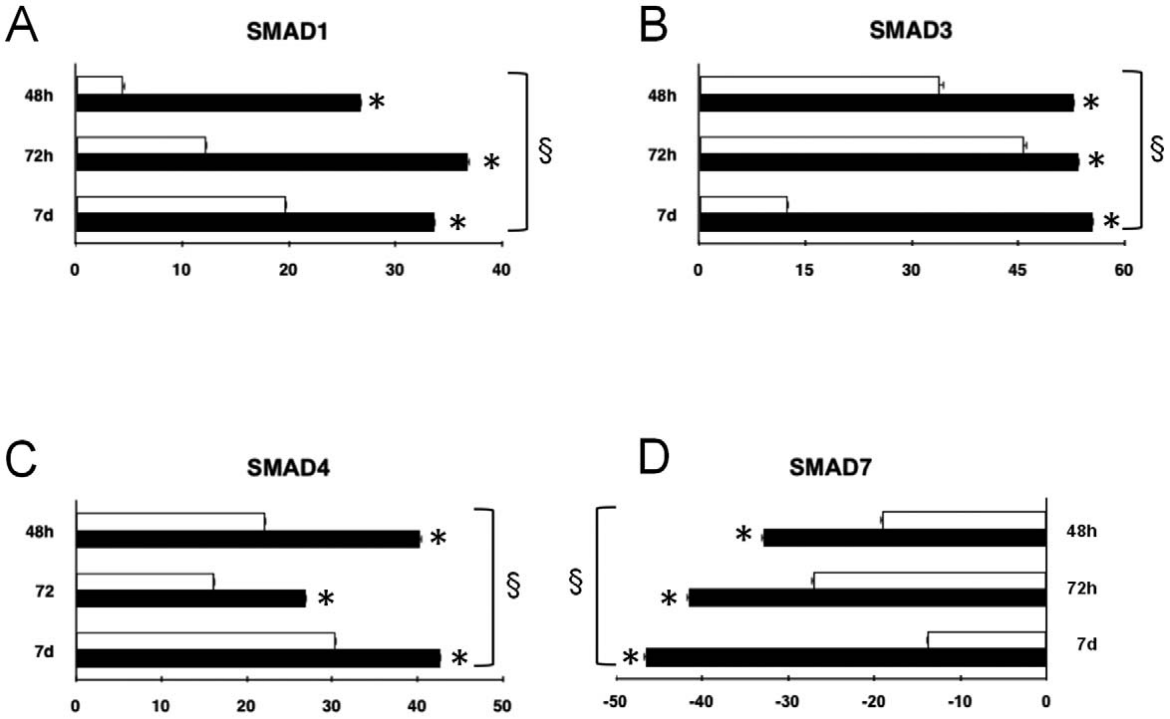
Effect of HBR on Smad gene expression in FMhMSCs. Cells were treated for 7 days in the absence (white bars) or presence (black bars) of HBR (1.5 mg/ml). For real-time RT-PCR analysis, the amount of each mRNA Smad1 (A), Smad3 (B), Smad4 (C) and Smad7 (D) was normalized to GAPDH and was plotted as fold change relative to the mRNA expression at time 0, defined as 1 (mean ± S.E.; n = 6). *, significantly different from untreated group;§, significant difference between groups (one-way analysis of variance, Tukey's test).

To further address whether the currently observed response to HBR may represent a general feature of this molecule in stem cell patterning, the effect of HBR on Smad gene expression was also investigated in FMhMSCs. We have previously shown that after exposure to HBR these cells exhibited a consistent increase in the transcription of the cardiogenic genes GATA-4 and Nkx-2.5, and differentiated into a high-yield of cardiac marker-expressing elements [5]. As shown in figure 2, FMhMSCs spontaneously expressed Smad1,3, and 4 mRNA. Interestingly, the gene expression of these Smads was significantly enhanced following a 48-hour exposure to the mixed ester, remaining upregulated during a subsequent period of 3 days, as compared with the untreated group (Figure 2A–2C). Confirming the results in HBR-treated GTR1 ES cells, FMhMSCs exposed to HBR displayed a lower amount of Smad7 mRNA, compared to unexposed cells (Figure 2D).

### HBR Affects Smad Protein Expression During the Cardiogenic Commitment in both GTR1 ES Cells and FMhMSCs

The cellular levels of Smad proteins result from a delicate balance between the rates of transcription of the corresponding genes and post-transcriptional regulatory mechanisms. Therefore, it was important to examine the expression of targeted Smad proteins in EBs and ES-derived cardiomyocytes exposed in the absence or presence of HBR.

**Figure 3:**
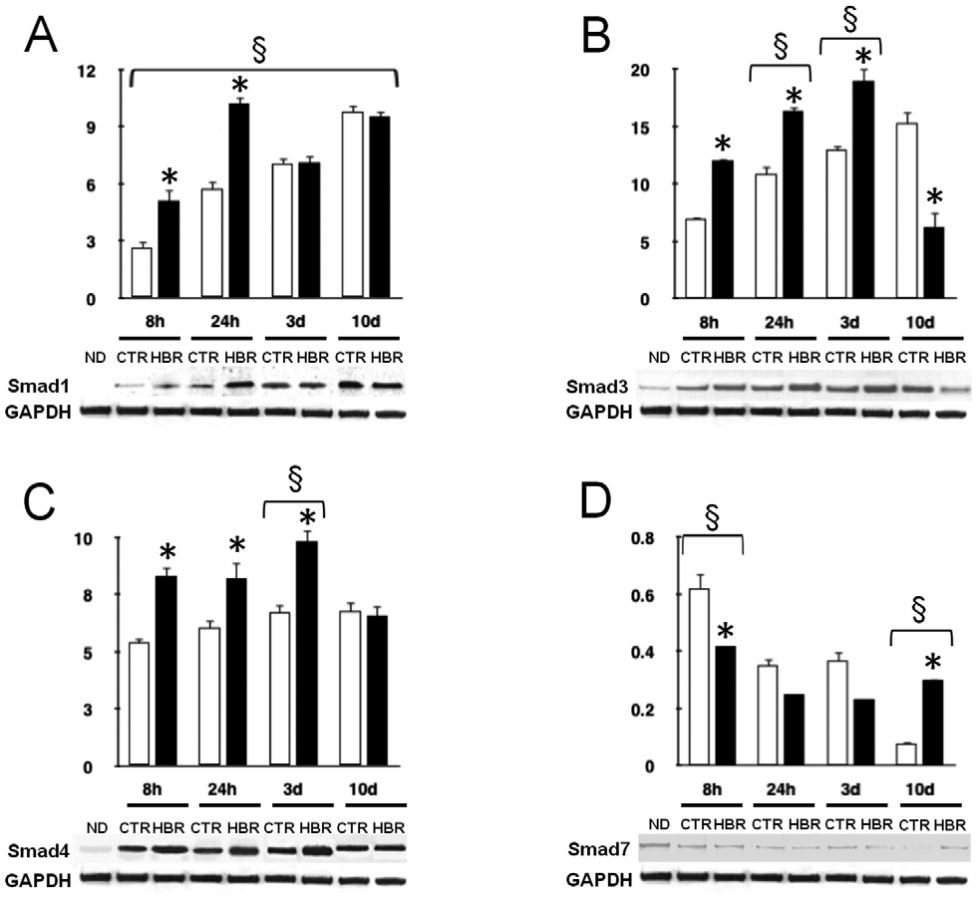
Effect of HBR on Smad protein expression during ES cells differentiation. Total lysates were obtained from undifferentiated GTR1 cells (ND) at the time of LIF removal (time 0), or from EBs (8 hours, 24 hours, and 3 days), or ES-derived puromycin selected cardiomyocytes (10 days) cultured in the absence (CTR, white bars) or presence (HBR, black bars) of 1.5 mg/ml HBR. Samples were subjected to Western blot analysis, using polyclonal antisera raised against Smad1 (A), Smad3 (B), Smad4 (C), and Smad7 (D). Sizes of the bands were determined with prestained marker proteins. Densitometric analysis was performed using Quantity one (BioRad). Data are reported relative to the expression in ND cells, and normalized to the expression level of GAPDH (mean ± S.E.; n = 6). *, significantly different from untreated group; §, significant difference between groups (one-way analysis of variance, Tukey's test).

As shown by Western blot and densitometric analyses, the spontaneous expression of Smad1 and 3 proteins, gradually increased after 8 hours, following LIF withdrawal (time zero), up to 10 days throughout ES cell commitment toward the cardiac phenotype (Figure 3A, 3B). During the same time-course Smad4 expression was already increased at 8 hours, with less appreciable changes in the subsequent time points (Figure 3C). HBR treatment differentially affected over time the expression of these Smad proteins, as compared with untreated cells, raising Smad1 at 24 hours (Figure 3A), while persistently enhancing the levels of Smad3 and 4 from 24 up to 72 hours (Figure 3B, 3C).

On the contrary, the expression of Smad7, which was consistently detectable in LIF-supplemented undifferentiated GTR1 cells (not shown), progressively declined during the cardiac differentiation timing. HBR further downregulated the expression of this Smad protein up to 72 hours, but increased it at the stage of ES-derived, puromycin selected cardiomyocytes (10 days), as compared to unexposed cells (Figure 3D).

**Figure 4:**
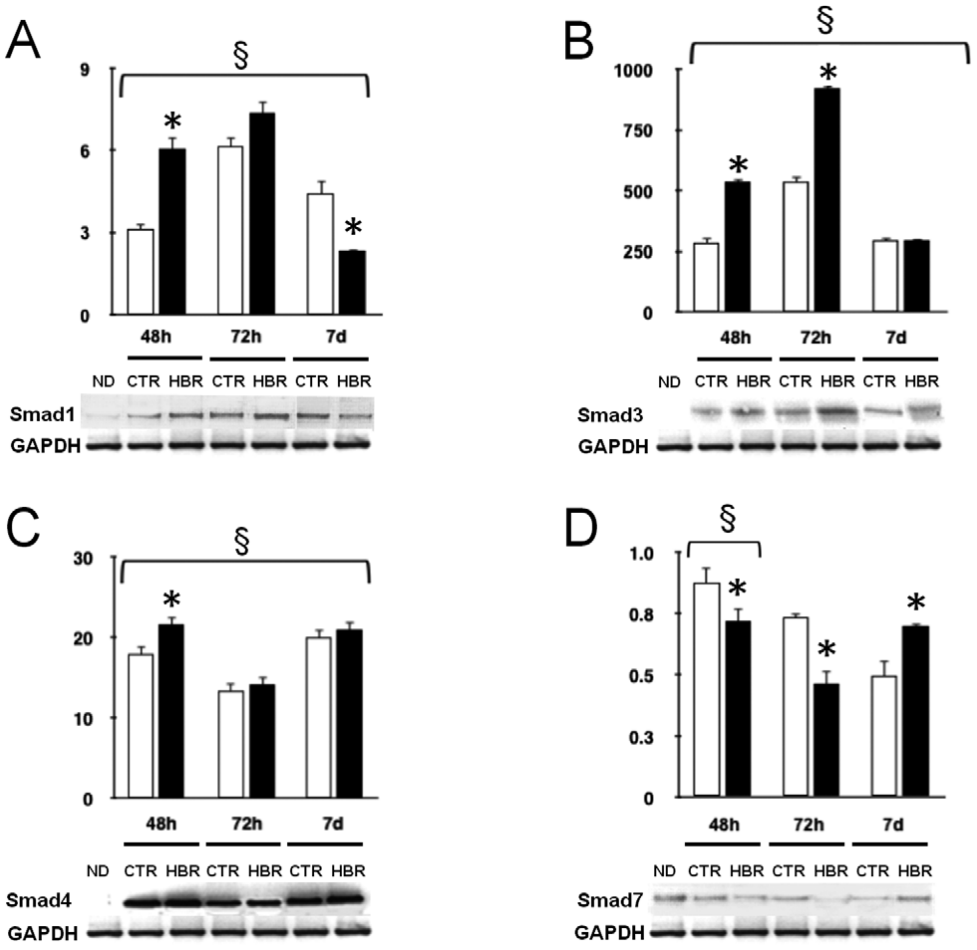
Effect of HBR on Smad protein expression during the differentiation of FMhMSCs. Total lysates were collected at time 0 (ND), considered as the time at which cells from the 3^rd^ passage reached 80% confluence, or from FMhMSCs cultured for different periods of time in the absence (CTR, white bars) or presence of 1.5 mg/ml HBR (HBR, black bars). Samples were analyzed by Western blot, using polyclonal antisera against Smad1 (A), Smad3 (B), Smad4 (C), and Smad7 (D). Sizes of the bands were determined with prestained marker proteins. Densitometric analysis was performed using Quantity one (BioRad). Data are reported relative to the expression in ND cells, and normalized to the expression level of GAPDH (mean ± S.E.; n = 6). *, significantly different from untreated group; §, significant difference between groups (one-way analysis of variance, Tukey's test).

Consistent with the observations in GTR1 ES cells, HBR significantly enhanced the protein expression of Smad1,3, and 4 in FMhMSCs, compared with untreated cells (Figure 4A–4C). The HBR effect on Smad4 expression was already evident after 48 hours, while a maximal upregulation of Smad1, and 3 was achieved after 72 hours of treatment. As in GTR1 cells, HBR accentuated the decline of Smad 7 expression observed up to 72 hours, but significantly counteracted the downregulation of this Smad at a later time (Figure 4D).

**Figure 5:**
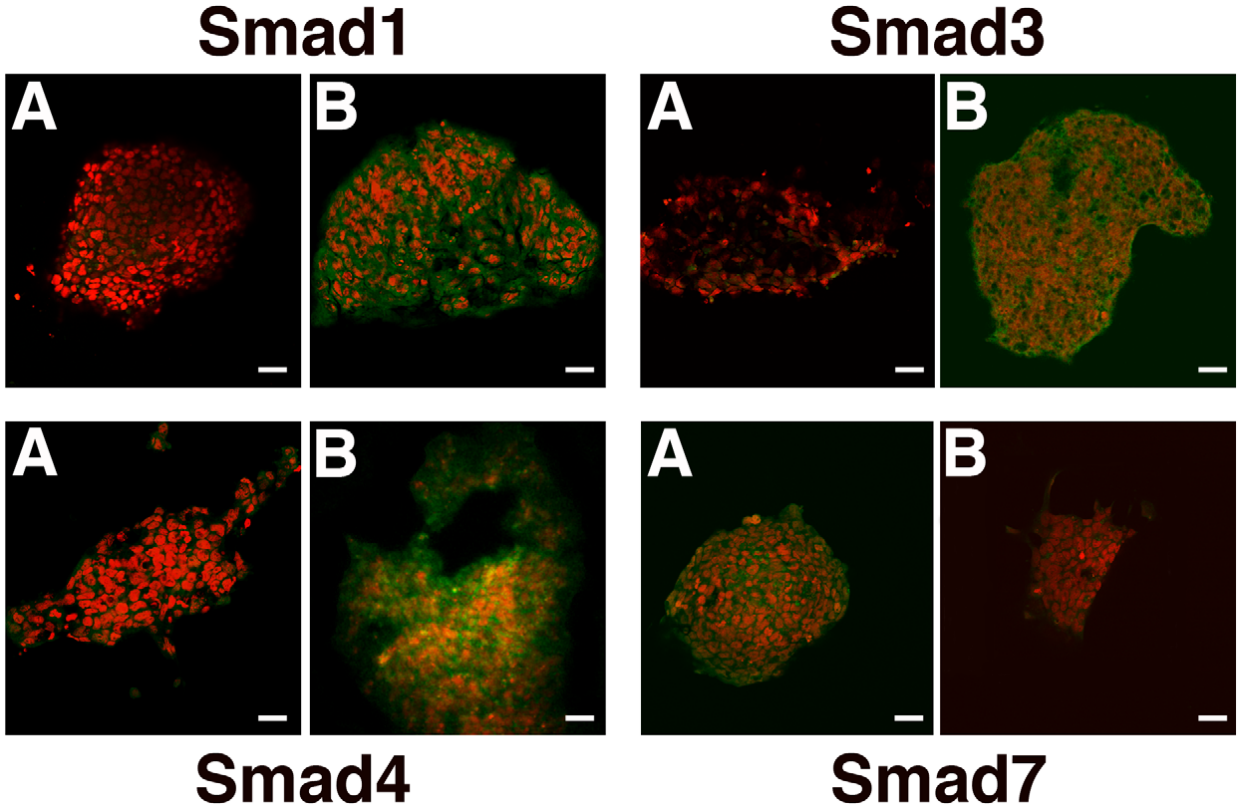
HBR modulates Smad protein expression in intact GTR1 ES cells. Smad1,3,4, and 7 protein expression was assessed in cells aggregated for 24 hours as EBs and cultured for additional 24 hours in the absence (A) or presence (B) of HBR (1.5 mg/ml). Scale bars are 40 µm. Nuclei are labeled with Propidium Iodide (*red*). Representative of five separate experiments.

We then assessed whether results from Western blot analysis may reflect the ability of HBR to regulate the intracellular patterning of targeted Smad proteins at the intact cell level. To this end, immunofluorescence analysis was performed in GTR1 ES cells, committed toward cardiac lineage in the absence or presence of the mixed ester. As shown in figure 5, HBR elicited a remarkable increase in the fluorescent staining for Smad1,3, and 4 (*panel B*), when compared to unexposed GTR1 ES cells (*panel A*). These observations further confirm that the stimulatory effect produced by HBR on the expression of Smad1,3, and 4 genes encompassed the upregulation of the encoded Smad proteins.

Immunofluorescence analysis also confirmed that HBR failed to induce the expression of Smad7 protein, as inferred from the greater fluorescence intensity detected in untreated versus HBR-exposed cells (Figure 5).

**Figure 6:**
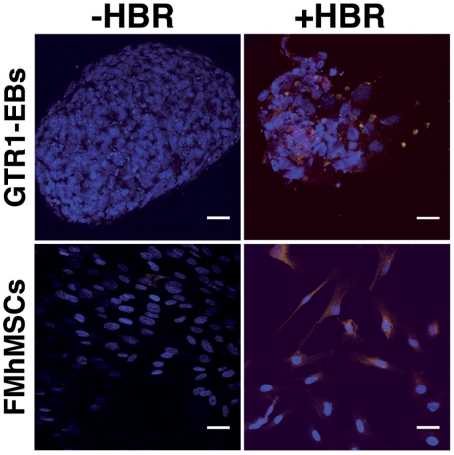
Immunofluorescence analysis of the expression of α-sarcomeric actinin and Smad4 in GTR1 ES cells and FMhMSCs. Concomitant expression of α-sarcomeric actinin [(FITC) *green* immunofluorescence] and Smad4 [(rhodamine) *orange* immunofluorescence] was assessed in GTR1-derived EBs or FMhMSCs cultured respectively for 3 or 7 days in the absence or presence of 1.5 mg/ml HBR. Scale bars are 40 µm. Nuclei are labeled with DAPI (*blue*). Representative of five separate experiments.

Interestingly, in separate experiments, we show that GTR1 EBs treated for 24 hours with HBR concomitantly expressed consistent amounts of both Smad4 and α-sarcomeric actinin, a marker of stem cell commitment to the cardiac lineage (Figure 6B). Otherwise, in untreated embryoid bodies the expression of Smad4 was only associated with a faint staining for α-sarcomeric actinin (Figure 6A). Akin to these observations, exposure of FMhMSCs for 7 days in the presence of HBR markedly enhanced the yield of Smad4 positive cells coexpressing α-sarcomeric actinin (Figure 6D), as compared to unexposed cells (Figure 6C).

### HBR Increases Smad4 Transcription in Isolated Nuclei

**Figure 7:**
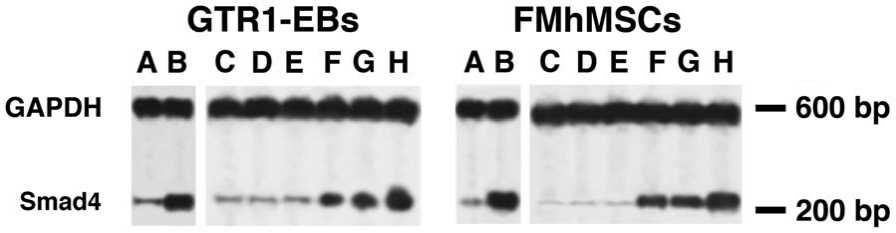
Analysis of Smad4 gene transcription in isolated nuclei. Nuclei were isolated from GTR1-derived EBs or FMhMSCs cultured for 24 hours in the absence (A) or presence (B) of 1.5 mg/ml HBR. From lanes C through H, nuclei were isolated from untreated cells and then directly incubated for 12 hours without any drug (C) or in the presence of 2.0 mg/ml HBR (D), 1.5 mg/ml hyaluronic acid (HA) (E), 2.5 mM butyric acid (BU) (F), 10^−8^M retinoic acid (RA) (G), or with a combination of BU and RA (H). Autoradiographic exposure was for 2 days on Kodak X-Omat film with an intensifying screen. The right side of each panel reports the position of radiolabeled DNA markers showing that the single protected fragments migrated with a molecular size comparable to Smad4 (268 bases), or GAPDH (597 bases) mRNA. Autoradiograms are representative of six separate experiments.

To further dissect the cellular response to HBR, nuclear run-off experiments were performed to assess whether HBR may have affected the rate of Smad4 gene transcription and whether, in the affirmative, it may have acted as a unit or after hydrolysis of its hyaluronan grafted moieties. Figure 7 shows that nuclei isolated from HBR-treated GTR1 ES cells or FMhMSCs exhibited a consistent increase in the transcription rate of Smad4, as compared with nuclei isolated from untreated cells. In separate experiments, nuclei were isolated from untreated cells and subsequently incubated with HBR, or exposed to HA, BU, or RA administered alone or in combination. While nuclear incubation with HBR or HA was ineffective, direct nuclear treatment with BU or RA enhanced Smad4 gene transcription (Figure 7). The transcription rate was further enhanced when nuclei were exposed to a combination of BU and RA (Figure 7).

### Nkx-2.5 Gene Expression During HBR-induced Cardiogenesis is Smad4-mediated

**Figure 8:**
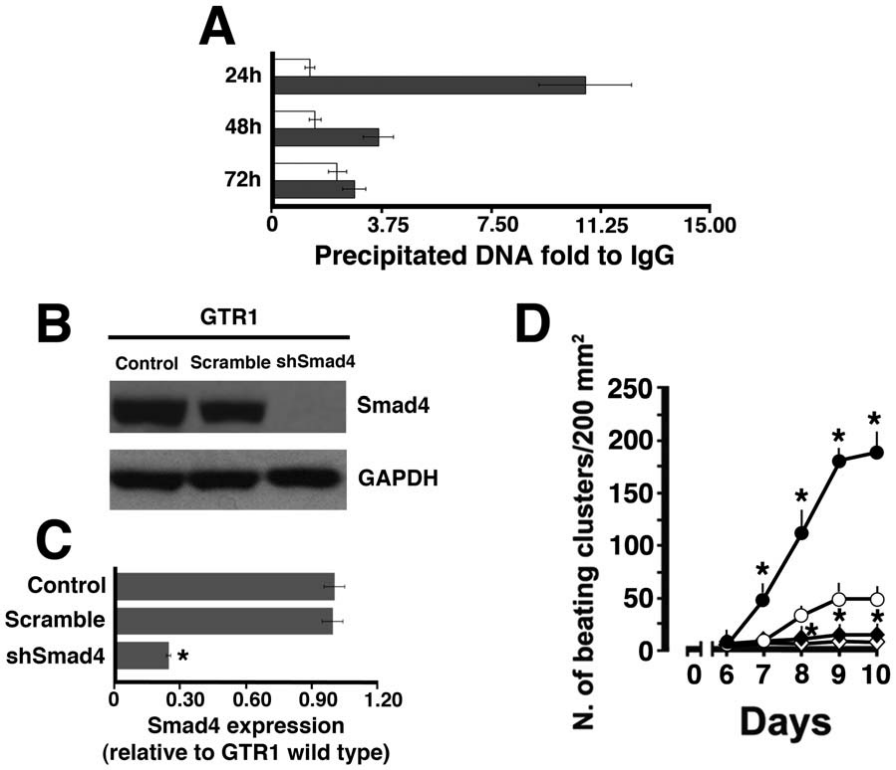
Smad4 binding to the Nkx-2.5 gene is crucial for HBR-mediated cardiogenesis. (A) ChIP was performed with Smad4 antibody on chromatin obtained from GTR1 ES cells, cultured in the absence (Control) or presence of 1.5 mg/ml HBR (HBR), at several hours of differentiation. The precipitated DNA was amplified by real-time PCR using specific primers for Nkx-2.5 promoter. Input DNA was used as reference and normal IgG immunoprecipitated DNA as calibrator. (B) Lentiviral-mediated Smad4 shRNA led to consistent silencing of Smad4 protein expression in GTR1 ES cells. A scrambled shRNA was used as a negative control. Samples were subjected to Western blot analysis, using polyclonal antisera raised against Smad4. Sizes of the bands were determined with prestained marker proteins. (C) Real-time RT-PCR analysis of Smad4 mRNA expression in GTR1 ES cells transduced in the absence (control, wild type) or presence of Smad4 or scrambled shRNA. Data are reported relative to the expression in GTR1 wild type cells (mean ± S.E.; n = 6). *, significantly different from wild type. (D) Analysis of the yield of puromycin-selected beating colonies obtained from wild type GTR1 ES cells or cells subjected to Smad4 silencing by the aid of lentiviral-mediated Smad4 shRNA. From the time of LIF removal throughout 4 days of puromycin selection, both groups of cells were treated in the absence (open circles and diamonds, wild type and Smad4-silenced, respectively) or presence of 1.5 mg/ml HBR (filled circles and diamonds, wild type and Smad4-silenced, respectively) (mean ± S.E.; n = 6). *, significantly different from untreated wild type cells.

To examine whether Smad4 was a transcription factor crucial in the HBR-mediated molecular program of cardiogenesis we performed chromatin immunoprecipitation (ChIP) analysis on ES cells treated in the absence or presence of HBR. We found that HBR-treated EBs exhibited a Smad4-binding on the promoter region of Nkx-2.5 gene (Figure 8A). Immunoprecipitation with a Smad4 antibody revealed that nuclear extracts from control and HBR-treated EBs generated an amplified product for the SBE binding site on the Nkx-2.5 promoter. Moreover, the amount of amplified DNA was significantly higher in HBR-treated GTR1 EBs, as compared to untreated controls (Figure 8A). The input control revealed that similar amounts of DNA were present in both untreated and HBR-stimulated EBs. To establish whether this mechanism may have a causal role in HBR-induced cardiogenesis, we performed a comparative analysis of the effect of HBR on the yield of ES derived cardiomyocytes in wild type GTR1 cells and GTR1 cells subjected to Smad4 silencing using lentiviral-mediated Smad4 shRNA, an approach that led to consistent silencing of Smad4 protein and mRNA expression (Figure 8B, 8C). Both groups of cells were cultured in the absence or presence of HBR from the time of LIF removal throughout 4 days of puromycin selection. Confirming our previous studies [4], GTR1 cell exposure to HBR resulted into a consistent increase in the number of spontaneously beating colonies, as compared with untreated controls (Figure 8D). Highlighting the important role of Smad4 in cardiac differentiation, Smad4 silencing nearly abrogated ES cell cardiogenesis, leading to a remarkable decline in the number of puromycin-resistant, spontaneously beating cells, representing only 1 to 2% of the cell population yielded from GTR1 wild type cells (Figure 8D). Interestingly, HBR failed to affect the amount of the few contracting aggregates spontaneously yielded in Smad4-silenced cells (Figure 8D).

## Discussion

Stem cell fate is controlled by multiple intrinsic regulators and by the extracellular matrix context. Under appropriate cell culture conditions, stem cells can spontaneously differentiate into various cell types, including cardiomyocytes. Nevertheless, spontaneous cardiogenesis is generally inefficient even in ES cells, occurring within a context of differentiated and undifferentiated cells, which are not suitable for cell therapy approaches and complicate efforts for the molecular dissection of differentiating programs. The *ex vivo* expansion and differentiation of both ES and adult multipotent stem cells are usually handled by cocktails of growth factors and genetic manipulation. Therefore, more efficient and selective methods are needed to drive targeted signaling orchestrator(s) of lineage commitment in stem cells, avoiding the cumbersome use of viral-mediated gene transfer technologies that are not readily envisionable in clinical practice.

There is compelling evidence that the Smad pathway is necessary for both heart development *in vivo* and cardiomyocyte differentation from pluripotent stem cells. Here, we sought evidence that HBR, a cardiogenic agent in both mouse ES cells and hMSCs [4], [5] that has recently shown to afford myocardial survival and repair even without stem cell transplantation [22], is able to finely regulate the molecular circuitry related to the Smad group of signal transducers. The finding that HBR concomitantly overexpressed Smad1 and 4 at both gene and protein level in GTR1 ES cells as well as in FMhMSCs is particularly rewarding. In fact, cooverexpression of Smad1 and 4, but not the transfection with either Smad1 or 4 alone, fully restored the process of cardiogenesis in P19CL6noggin, a line which constitutively overexpresses the BMP antagonist noggin loosing the ability to differentiate into cardiomyocytes [23]. The current observations indicate that the Smad1/4 system can be efficiently activated by HBR without the needs of BMP stimulation. Moreover, the finding that HBR-mediated Smad1/4 overexpression involved the differentiation in cardiac marker expressing cells of both GTR1 ES cells and FMhMSCs indicates that the mixed ester was able to couple the activation of Smad signaling with the execution of a program of cardiac lineage specification.

The functional implications of HBR-induced Smad3 gene and protein expression remain to be clarified. However, it has been shown that upon R-Smad phosphorylation, Smad3 may have a pivotal role in the continuation of the signaling cascade being involved in the assembly of Smad4 into a heterotrimeric complex with a stoichiometry of two Smad3 subunits to one Smad4 subunit [24]. HBR-mediated increase in Smad1 protein expression was short-lived compared to the mixed ester effect on Smad3 and 4. Recently, it has become evident that Smad levels are subjected to complex, differential regulation by selected players within the ubiquitination-proteasome pathway [25]. We cannot exclude that differences in time-course response of individual Smads to HBR may reflect inherent changes in these still largely unexplored patterning of post-transcriptional modifications. Additional work is needed to clarify the possible implications of the HBR effect on the carefully regulated interplay of Smad members.

The downregulation by HBR of gene and protein expression of Smad7, an inhibitory Smad, may represent a relevant part of the cardiogenic effect of the mixed ester. In fact, Smad7 gene expression has been found to be potently increased by LIF, a cytokine capable of maintaining ES cells in an undifferentiated state [9]. It is also evident that Smad7 blocks the activation of R-Smads, and/or competes with activated R-Smads for heteromeric complex formation with Smad4 [26], [27], abrogating cardiac differentiation in pluripotent cells [23]. Hence, the cardiogenic action of HBR may have been enhanced by its ability to suppress an autoregulatory negative feedback loop exerted by inhibitory Smads during the early period of Smad1/4 upregulation. On the other hand, it has been recently shown that Smad7 deficient mice died *in utero* due to multiple defects in cardiovascular development, suggesting that Smad7 has an important role in development and function of heart *in vivo* [28]. Moreover, long-term treatment with B-type natriuretic peptide significantly attenuated cardiac hypertrophy via the Smad7 pathway *in vivo* and *in vitro* [29]. These findings indicate that once the cardiac phenotype is established, Smad7 may have an important role in preventing myocardial remodeling at both tissue and cardiomyocyte level. Intriguingly, while HBR decreased Smad7 gene and protein expression in EBs, during the maximal induction of Smad1/4, it reversed its action counteracting the spontaneous decline of the Smad7 protein expression at a later period when cardiomyocytes were formed from GTR1 ES cells or a cardiac-like phenotype was established from FMhMSCs. These observations suggest that HBR, by finely regulating the balance of different Smads, may also control cell growth homeostasis throughout the initial cardiogenic commitment and the terminal lineage specification.

Mechanisms underlying HBR-mediated changes in Smad gene expression remain to be fully elucidated. We have previously shown that the ability of HBR to increase the expression of a number of genes enrolled in stem cell survival and/or cardiovascular commitment involves a direct action of HBR-grafted moieties, particularly BU and RA, on the nuclear transcriptional machinery [5], [22]. Akin to these observations, nuclear run-off experiments revealed that the increase in Smad4 mRNA elicited by HBR was mediated at the transcriptional level, and could be reproduced with additive effects by a direct exposure of isolated undifferentiated nuclei to BU and RA, but not to the intact mixed ester. These findings prompt the hypothesis that, at least at nuclear level, HBR may have acted following the hydrolysis of its grafted moieties. Within this context, we have recently shown that HBR is able to enhance histone acetylation in both infarcted rat hearts and isolated rat cardiac myocytes and Stro-1 positive stem cells [22]. Consonant with the current observations, Smad transcription and protein expression have been reported to be largely affected through chromatin remodeling induced by BU and other HDAC inhibitors [30]–[32]. An inference of the retinoid moiety of HBR in Smad4 transcription is consistent with previous findings showing that retinoic acid activation of BMPR/Smad transcription and signaling is an important molecular trait in a number of differentiating processes [33]–[36]. Moreover, RXR/RAR heterodimer action is enhanced by histone deacetylase inhibitors, promoting major developmental pathways in pluripotent cells [37]. Studies are in progress to further elucidate the trascriptional and nuclear signaling profile(s) coupled with the wide spectrum of Smads in response to the mixed ester.

We have previously shown that both in mouse ES cells and hMSCs isolated from different sources the cardiogenic action of HBR encompassed an increase in the gene expression of Nkx-2.5 [4], [5]. It is now evident that in early heart progenitor cells the Smad patterning converges to the binding of Smad4 to a highly conserved Smad site in the Nkx-2.5 cardiac enhancer and that this mechanism is central for the entire cardiogenic process. The N-terminal, Mad homology-1 domain of Smad4 binds DNA via a Smad binding element (SBE) identified as AGAC [18]. The presence of a Smad regulatory element in the 5′flanking region of the mouse Nkx-2.5 gene has also been described [38]–[42].

The current ChIP experiments show that nuclear extracts from control and HBR-treated EBs generated an amplified product for the SBE binding site on the Nkx-2.5 promoter and that the amount of amplified DNA yielded from nuclei extracted from HBR-treated ES cells was significantly higher than that derived from control cell nuclei. These findings provide evidence that a synthetic molecule can be used to efficiently enhance Smad4 binding on Nkx-2.5 gene. The observation that in GTR1 cells subjected to Smad4 silencing HBR completely lost its ability to enhance the formation of spontaneously beating ES-derived cardiomyocytes indicates that HBR-mediated recruitment of Smad4 to the Nkx-2.5 gene is a mandatory step in the execution of the cardiogenic program driven by the mixed ester. Assessing the consequences of HBR-induced increase in Nkx-2.5 gene expression and Smad patterning in hMSCs will require further investigation to establish whether these cells may exhibit electrophysiological features and contractile activity of fully compliant cardiomyocytes. Nevertheless, we have shown that HBR-treated FMhMSCs retained *in vivo* a cardiac-like phenotype when transplanted in infarcted rat hearts, remarkable enhancing cardiac repair, as compared with hearts transplanted with untreated cells [5].

In conclusion, the clinical use of stem cells will be hampered in a near future by a number of interrelated challenges, including: (i) high-throughput bioprocess development and improved downstream processing problems; (ii) significant modification, improvement and re-testing of current strategies of stem cell culturing and lineage commitment complying with all standards of Good Manufacturing Practice (GMP); (iii) the development of new chemistry to maximize differentiation efficiencies. Within this context, we have developed HBR, a synthetic compound affording a chemical manipulation of one of the major family of signal transducers, optimizing stem cell cardiogenesis through a fine tuning of Smad transcription and signaling without the needs of viral vector mediated gene transfer technologies.

## Materials and Methods

### ES Cells

GTR1, a derivative of R1 ES cells bearing the puromycin-resistance gene driven by the cardiomyocyte-specific α-myosin heavy chain (MHC) promoter, were kindly provided by Dr. W.L. Stanford (University of Toronto, Canada). GTR1 cells were maintained in the undifferentiated state by culturing onto a layer of mitotically inactivated mouse embryo fibroblasts in the presence of Knockout DMEM containing 15% fetal bovine serum, supplemented with a final concentration of 1000 U/ml Leukemia Inhibitory Factor (LIF). Cell differentiation and puromycin-mediated selection of ES-derived cardiomyocytes were performed as previously described [4]. Briefly, cells were plated onto specialty plates (Costar ultra low attachment clusters), containing the culture medium lacking supplemental LIF. After 2 days of culture, the resulting embryoid bodies (EBs) were plated onto tissue culture dishes. When spontaneous contractile activity was noticed (7 days after LIF removal), puromycin (2 µg/ml) was added to eliminate non-cardiomyocytes, and puromycin-selected cells were cultured for an additional period of 4 days.

### Lentivirus Production and Infection of GTR1 ES Cells

The packaging cells (293FT cell line, Invitrogen, Cat. N. R700-07) were seeded at 1.5×10^5^ cells/ml (6 ml per plate) in low-antibiotic growth media (DMEM +10% FBS) in 6 cm tissue culture plates. After 24 hours, the packaging cells were transfected with 3 lentivirus plasmids. 1 µg of Hairpin- pLKO.1 vector (Smad4 shRNA, Sigma), 0.9 µg of packaging plasmid psPAX2, and 0.1 µg of envelope plasmid pMD2G were diluted in OPTI-MEM to a total volume of 250 µl. 24 µl of FuGene HD were added to the plasmids mix and incubated 20 minutes at room temperature. The transfection mix was transferred to the packaging cells. The cells were incubated at 37°C, 5% CO_2_. 18 hours post-transfection, the medium was replaced with fresh high-serum medium. After 24 hours, the viruses in the medium were harvested and then replaced with high-serum media. Twenty-four hours after the first harvest, the virus was harvested and the packaging cells were discarded. The media containing virus was filtered with 0.45 µm filter. The eluate was transferred to a sterile polypropylene storage tube. GTR1 cells were infected with 1 ml (1 MOI) of virus solution. After 18–24 hours of incubation, the media were replaced with growth media. Real-time RT PCR was used to assess the downregulation of Smad4 mRNA expression with the following primers: Smad4 forward: GGACGACTTCAGGTGGCTG; Smad4 reverse: CCTGAGAGATCAATTCCAGGTG. Smad4 protein expression was also determined by Western blot analysis.

### FMhMSCs

FMhMSCs were isolated as described [5]. Briefly, term placenta obtained from caesarian sections were rapidly rinsed in PBS containing penicillin and streptomycin and used immediately. Pieces from fetal membranes were minced and digested for 10 minutes in DMEM with 0.25% trypsin, 10 U/ml DNaseI and 0.1% collagenase. Tissues were pipetted vigorously up and down avoiding foam for 5 minutes; larger pieces of tissue were allowed to settle under gravity for 5 minutes. Each supernatant was transferred to a fresh tube, neutralized with FBS, then spun at 1000× g for 10 minutes. Each pellet was resuspended in 5 ml of DMEM containing 20% FBS, 10 U/ml penicillin and 100 µg/ml streptomycin. FMhMSCs were seeded into 25-cm^2^ flasks and grown at 37°C in 5% CO_2_. Non-adherent cells were removed after 1 week and medium (with 10% FBS) was changed subsequently every four days.

### Gene Expression

Total RNA was isolated using Trizol reagent according to the manufacturer's instruction (Invitrogen). Total RNA was dissolved in RNAase-free water and, for RT-PCR, cDNA was synthesized in a 50-µl reaction volume with 1 µg of total RNA and MuMLV reverse transcriptase (RT) according to the manufacturer's instruction (Invitrogen). Quantitative real-time PCR was performed using an iCycler Thermal Cycler (Bio-Rad). Two µl cDNA were amplified in 50-µl reactions using Platinum Supermix UDG (Invitrogen), 200 nM of each primer, 10 nM fluorescein (BioRad), and Sybr Green. After an initial denaturation step at 94°C for 10 min, temperature cycling was initiated. Each cycle consisted of 94°C for 15 s, 55–59°C for 30 s and 60°C for 30 s, the fluorescence being read at the end of this step. All primers used in this study were from Invitrogen and previously described by other Authors [43], [44]. To evaluate the quality of product of real-time PCR assays, melting curve analysis was performed after each assay. Relative expression was determined using the “delta-CT method” with GAPDH as reference gene [45].

### Nuclear Run-off Transcription Assay

Isolation of nuclei and assessment of nuclear purity were performed as detailed elsewhere [5], [22]. Only freshly isolated nuclei were used in each experiment. Nuclear run-off experiments were carried out as previously described [5], [22]. Nuclear RNA was isolated by using guanidine thiocyanate and acid phenol extraction, followed by purification on RNAMATRIX™. Equal counts of ^32^P-labeled RNA (about 5·10^6^ cpm) were then subjected to a solution hybridization RNase protection assay and were hybridized for 12 hours at 55°C in the presence of unlabeled antisense Smad4 mRNA. To generate these cRNA probes, cDNA fragments of rat Smad4 (268 bp), or GAPDH (597 bp) genes were inserted into a pCRII-TOPO vector. Transcription of plasmids linearized with BamHI generated antisense strands of GAPDH mRNA, whereas transcription of plasmids linearized with EcoRI produced an antisense strand of Smad4 mRNA. Samples were then incubated with a combination of RNase A and T1 and exposed to proteinase K. The protected fragments were recovered after phenol chloroform extraction and electrophoretically separated in a polyacrylamide non-denaturing gel. Autoradiographic exposure was for 48 hours.

### Immunoblotting analysis

ES cells and FMhMSCs were collected in PBS, than pellets were lysates with cell extraction buffer (Invitrogen). Total cell lysates, from GTR1 ES cells and from FMhMSCs were electrophoresed on 10% Novex Tris-glycine polyacrylamide gels (Invitrogen, CA), in MOPS SDS Running Buffer, using an XCell SureLock™ Mini-Cell, according to the instruction provided by the manufacturer. After protein transfer to polyvinylidene difluoride (PVDF) membranes (Invitrogen, CA), membrane saturation and washing, the immunoreaction was carried out for 1 hour at room temperature in the presence of the primary antibody (antisera against Smad1, Smad3, Smad4, and Smad7) (AbCAM) diluted 1∶1000. After additional washing, membranes were incubated with anti-rabbit horseradish peroxidase (HRP) conjugated secondary antibody (AbCAM). Targeted Smad expression was assessed by a chemioluminescence detection system (ECL Western blotting detection reagents were from Amersham Biosciences).

### ChIP Analysis

Cells were plated in ultralow attachment clusters plates (Costar) in growth medium without LIF. After treatment with HBR, cells were collected at 24, 48, and 72 hours. ChIP analysis was carried out with the Upstate Biotechnology's ChIP Assay Kit (Upstate Group), following manufacturer's instructions. After fixation with 1% formaldehyde (Sigma, St. Louis, MO) for 20 min at room temperature, cells were collected and lysed in ice for 10 minutes. Lysates were then sonicated at amplitude 10% in eleven cycles of 30 s, spaced out 15 s, using a Fisher Model 550 Sonic Dismembrator (Fisher, Pittsburgh, PA). ChIP was performed overnight using 2 µg of either normal rabbit IgG or anti-Smad4 (Santa Cruz). Following the elution, samples were phenol-chloroform extracted and resuspended in nuclease-free water. Real-time PCR was performed using the Applied Biosystem 7300 System with a FastStart Universal SYBR Green Master (ROX) (Roche). Data from real-time PCR experiments were analyzed with the “delta-CT method” [45]. Each data point was obtained from three independent experiments. Input DNA (1% of total chromatin used for the immunoprecipitation reactions) was used as reference and the background signal (normal IgG immunoprecipitated DNA) as calibrator. The sequences of the primers used are: Nkx2.5 forward ACAGAAACCCCCATCTGTTTCC; Nkx2.5 reverse CTGCAATCAGCCGCGAAAAGTA. Final values shown here are fold differences relative to the background, obtained with the formula:

_2_[(Ct background – Ct Input background) – Ct Ab – CT Input Ab)]

where Ct is the threshold cycle, background is the normal rabbit IgG immunoprecipitated sample, and Ab is the Smad4 immmunoprecipitated sample. To ensure specific PCR amplification, every real-time PCR run was followed by a dissociation phase analysis (denaturation curve).

### Immunostaining

Puromycin-selected cells were treated with trypsin, and the resulting suspension was cultured at low density to permit visualization of individual cells. The cultures were fixed with 4% paraformaldehyde. Cells were exposed for 1 hour at 37°C to mouse monoclonal antibodies against α-sarcomeric actinin or Smad1, or with rabbit polyclonal antibodies against Smad3, Smad4, and Smad7, and stained at 37°C for 1 hour with fluorescein-conjugated goat IgG.

For the double staining immunofluorescence we used the parallel approach. Briefly, cells were simultaneously labeled for 1 hour at 37°C with anti α-sarcomeric actinin mouse monoclonal antibody and with anti Smad4 rabbit polyclonal antibody (1∶100). Cells were then stained with fluorescein-conjugated goat IgG and with rhodamine conjugated mouse anti-rabbit IgG. All microscopy was performed with a Leica confocal microscope (Leica TCSSP5). DNA was visualized with Propidium Iodide (1 ug/ml) or DAPI (1 µg/ml).

### Data analysis

The statistical analysis of the data was performed by using a one-way analysis of variance followed by Tukey's multiple comparison test, and assuming a *p* value less than 0.005 as the limit of significance.
